# Exclusive Breastfeeding Is Not Associated with Maternal–Infant Bonding in Early Postpartum, Considering Depression, Anxiety, and Parity

**DOI:** 10.3390/nu13041184

**Published:** 2021-04-02

**Authors:** Naoki Fukui, Takaharu Motegi, Yuichiro Watanabe, Koyo Hashijiri, Ryusuke Tsuboya, Maki Ogawa, Takuro Sugai, Jun Egawa, Takayuki Enomoto, Toshiyuki Someya

**Affiliations:** 1 Department of Psychiatry, Graduate School of Medical and Dental Sciences, Niigata University, Niigata 951-8510, Japan; fukui@med.niigata-u.ac.jp (N.F.); motegi@med.niigata-u.ac.jp (T.M.); yuichiro@med.niigata-u.ac.jp (Y.W.); konokome@yahoo.co.jp (K.H.); tenseatmosphere@yahoo.co.jp (R.T.); makifujita427@gmail.com (M.O.); tsugai@med.niigata-u.ac.jp (T.S.); jeg5414@med.niigata-u.ac.jp (J.E.); 2 Department of Obstetrics and Gynecology, Graduate School of Medical and Dental Sciences, Niigata University, Niigata 951-8510, Japan; enomoto@med.niigata-u.ac.jp

**Keywords:** breastfeeding, maternal–infant bonding, depression, anxiety, early postpartum

## Abstract

It is important to clarify how the breastfeeding method affects women’s mental health, and how women’s mental health affects the breastfeeding method in the early postpartum period when major depression and other psychiatric problems are most likely to occur. This study aimed to examine this bidirectional relationship in the early postpartum period. Participants were 2020 postpartum women who completed the Hospital Anxiety and Depression Scale (HADS) and Mother-to-Infant Bonding Scale (MIBS). We obtained data for participants’ breastfeeding method for four weeks after childbirth. We performed a path analysis with factors including breastfeeding method (exclusive breastfeeding or non-exclusive breastfeeding), parity (primipara or multipara), the two HADS subscales (anxiety and depression), and the two MIBS subscales (lack of affection and anger and rejection). The path analysis showed that breastfeeding method did not significantly affect depression, anxiety, and maternal–infant bonding in the early postpartum period. Women with higher anxiety tended to use both formula-feeding and breastfeeding. Our study suggests that exclusive breastfeeding is not associated with maternal-fetal bonding in early postpartum, considering depression, anxiety, and parity.

## 1. Introduction

The concept of maternal–infant bonding was popularized by Klaus and Kennell [1]. An important contention of their bonding theory is that close contact is necessary for establishing maternal–infant bonding. Other literature also describes breastfeeding as promoting bonding [2]. Furthermore, the World Health Organization (WHO) stated that “exclusive breastfeeding means that the infant receives only breast milk. No other liquids or solids are given—not even water—with the exception of oral rehydration solution, or drops/syrups of vitamins, minerals, or medicines.” The WHO recommends exclusive breastfeeding for the first six months of life, claiming benefits for fetal and maternal physical health as well as secure maternal–infant bonding [3]. However, few studies have scientifically investigated the relationship between breastfeeding method and maternal–infant bonding.

Cernadas et al. evaluated maternal–infant bonding during the first days after birth using a self-designed tool and obtained data for breastfeeding method up to six months from 539 mothers [4]. They reported that good mother–infant bonding predicted a longer duration of exclusive breastfeeding, after adjusting for confounding factors such as age, parity, and education level. Else-Quest et al. used the Parenting Stress Index, which includes a maternal–infant bonding scale, at 4- and 12-months postpartum with 570 mothers [5]. They reported that the exclusive breastfeeding group tended to show better mother–infant bonding at four months (although the relationship was not significant), after adjusting for confounding factors such as age and education level. Nishioka et al. compared degrees in depressive symptoms and maternal–infant bonding between a breastfeeding-based group (*n* = 278) and a formula milk-based group (*n* = 127) [6]. They reported the breastfeeding-based group showed better mother–infant bonding compared with the formula milk-based group. However, we found inconsistent results and critical methodological differences among these studies. For example, Cernadas et al. examined a directional relationship from maternal–infant bonding to breastfeeding [4], whereas Else-Quest et al. examined a directional relationship from breastfeeding to maternal–infant bonding [5]. In contrast, Nishioka et al. performed intergroup comparisons [6]. Therefore, it is necessary to perform a bidirectional analysis (i.e., from breastfeeding to maternal–infant bonding and vice versa). We used a path analysis to perform this bidirectional analysis, with consideration of latent confounding factors.

Depression is considered an important confounding factor when examining the relationship between breastfeeding and maternal–infant bonding. This is because several studies have shown depression had significant effects on maternal–infant bonding. We discussed the relationships between postpartum depression and maternal–infant bonding in our recent study [7]. In addition, several studies have noted a bidirectional relationship between depression and breastfeeding. Figueiredo et al. used the Edinburgh Postpartum Depression Scale (EPDS) from pregnancy to three months postpartum and collected self-report exclusive breastfeeding data from birth to 12 months postpartum (*n* = 145 women) [8]. They reported that depressive symptoms during pregnancy predicted early cessation of exclusive breastfeeding, and that exclusive breastfeeding predicted reduced symptoms of depression from childbirth to three months postpartum. The Norwegian Cohort Study (*n* = 42,225) assessed maternal depression symptoms at 30 weeks gestation and six months postpartum and collected data for breastfeeding methods at six months postpartum [9]. In that study, women with higher levels of depression stopped breastfeeding early compared with those with lower levels of depression, and breastfeeding cessation was a risk factor for increased depression. Based on these results, depression, maternal–infant bonding, and breastfeeding methods could influence each other. However, few studies have investigated the relationships among these three factors.

Postpartum anxiety has also been reported to be associated with both maternal–infant bonding and exclusive breastfeeding. We also considered the relationship between postpartum anxiety and maternal–infant bonding in our recent study [7]. Several studies have reported that women with postpartum anxiety tended to have difficulties starting breastfeeding and terminated breastfeeding early [10,11]. Consequently, we performed path analysis to clarify the relationships among breastfeeding methods, depression, anxiety, and maternal–infant bonding.

## 2. Materials and Methods

### 2.1. Ethics Statement

This study was part of the Perinatal Mental Health Research Project, which we conducted jointly with the Niigata University Medical and Dental Hospital, Department of Obstetrics and Gynecology, and 33 associated obstetric institutions in Niigata Prefecture, Japan. This study was approved by the Niigata University Ethics Committee (approval number: 2016–0019) and the ethics committees of the participating obstetric institutions. Written informed consent was obtained from all participants.

### 2.2. Participants

This study was part of the Perinatal Mental Health Research Project, which started in March 2017 [7,12,13]. We collected data for breastfeeding methods, the Japanese versions of the Hospital Anxiety and Depression Scale (HADS), and the Mother-to-infant Bonding Scale (MIBS) for 2020 postpartum women (1029 primipara and 991 multipara, mean age 32.2 ± 4.8 years) who visited the participating obstetric institutions at 1 month after giving birth. The participants were the same cohort as used in our previous study that reported the relationships between anxiety, depression, parity, and mother–infant bonding in 2379 Japanese mothers [7]. However, we excluded those without data for breastfeeding methods. Women with serious physical complications, serious pregnancy complications, or severe psychiatric disorders (e.g., severe schizophrenia or severe depression) were also excluded.

### 2.3. Measurements

In this study, self-completed questionnaires were consecutively used at three time points: early pregnancy (approximately 12–15 weeks), late pregnancy (approximately 30–34 weeks), and postpartum (4 weeks after childbirth).

The HADS is commonly used to screen for anxiety and depression in clinical settings [14] and was translated into Japanese by Kitamura [15]. The scale comprises 14 questions: seven items assess anxiety (items 1, 3, 5, 7, 9, 11, and 13), and seven assess depression (items 2, 4, 6, 8, 10, 12, and 14). Participants are asked to rate all items on a four-point Likert-type scale from 0 to 3.

The Mother–Infant Bonding Questionnaire (MIBQ) developed by Kumar and colleagues [16] is used to evaluate bonding disturbance during the postpartum period. Those authors revised the MIBQ to develop the MIBS [17]. Marks and colleagues further modified the MIBS by changing the wording of some items and adding a new item. This modified version of the MIBS was translated into Japanese (MIBS-J) by Yoshida et al. [18]. We described the MIBS-J in detail in our previous studies [7,12]. Participants are asked to rate the 10 MIBS-J items on a four-point Likert-type scale from 0 to 3. Our previous study detected a two-factor structure for the MIBS-J: lack of affection (items 1, 6, 8, and 10) and anger and rejection (items 2, 3, 5, and 7) [12]. Therefore, we used these two factors in this study.

Obstetric characteristics such as parity (primiparous or multiparous) and breastfeeding methods during the first four weeks after childbirth were obtained through interviews by midwives. This study was a sub-analysis of postpartum data up to December 2019.

### 2.4. Statistical Analysis

In this study, the numbers of women with exclusive breastfeeding, that added any formula-feeding to breastfeeding, and with formula-feeding only were 1029, 945, and 46, respectively.

First, we compared characteristics such as parity, age, and HADS and MIBS subscale scores among exclusive, mixed, and formula-feeding groups. We used chi-square tests for categorical data and analysis of variance (ANOVA) for continuous variables. The level of significance was set at *p* < 0.0063 according to the Bonferroni correction of eight statistical tests. We used the Bonferroni post hoc test following the ANOVA and adjusted residual after chi-square test.

Second, we performed path analysis for factors, including breastfeeding method, parity, the two HADS subscales (anxiety and depression), and the two MIBS subscales (lack of affection and anger and rejection). For the breastfeeding method, we divided our participants into an exclusive breastfeeding group and a non-exclusive breastfeeding group, with the latter comprising the mixed and formula-feeding groups.

We used the root mean square error of approximation (RMSEA) as the index to evaluate the goodness of fit between the models and the data.

All statistical analyses were conducted using SPSS version 25 (IBM Corp., Armonk, NY, USA) and Amos 25.0.0 (IBM Japan, Tokyo, Japan).

## 3. Results

Table 1 shows comparisons of characteristics among the exclusive, mixed, and formula-feeding groups. There was a significant difference in the ratio of primiparas to multiparas among the three groups (*p* < 0.001). Frequency of primipra was significantly higher in mixed group that exclusive group. Mean age did not significantly differ among the groups (*p* = 0.012). HADS anxiety scores were significantly higher in the mixed group (6.08 ± 4.07 vs. 4.92 ± 3.60, *p* < 0.001) and formula-feeding group (6.57 ± 4.25 vs. 4.92 ± 3.60, *p* = 0.014) than in the exclusive group. HADS depression scores were significantly higher in the mixed group (6.59 ± 3.44 vs. 5.87 ± 3.16, *p* < 0.001) than in the exclusive group. Additionally, MIBS lack of affection scores were significantly higher in the mixed group (1.02 ± 1.30 vs. 0.73 ± 1.09, *p* < 0.001) than in the exclusive group. MIBS anger and rejection scores also were significantly higher in the mixed group (0.46 ± 0.94 vs. 0.31 ± 0.73, *p* < 0.001) than in the exclusive group.

**Figure 1:**
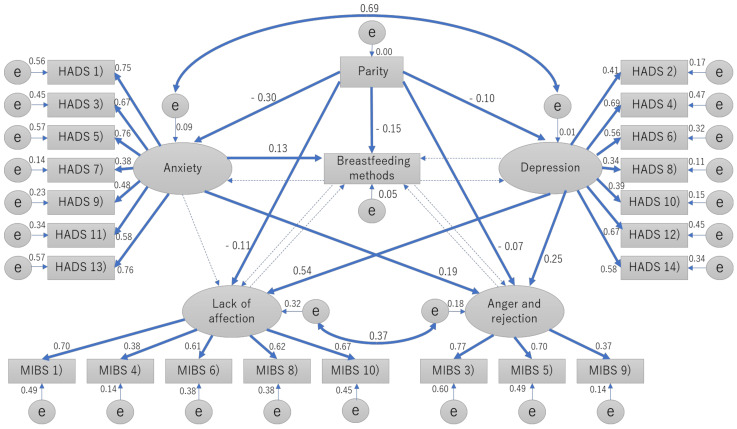
Path model of the associations between breastfeeding methods, parity, depression, anxiety, and maternal–infant bonding in the early postpartum period. Significant paths are shown in bold. Abbreviations: HADS, Hospital Anxiety and Depression Scale; MIBS, Mother-to-Infant Bonding Scale.

Figure 1 shows the path model that describes interactions between the two HADS subscales and the two MIBS factors with a good model fit [19] to the data (RMSEA = 0.052). Being primipara predicted non-exclusive breastfeeding (*p* < 0.001, r = −0.15), anxiety (*p* < 0.001, r = −0.30), depression (*p* < 0.001, r = −0.10), lack of affection (*p* < 0.001, r = −0.11), and anger and rejection (*p* = 0.003, r = −0.07). Anxiety predicted non-exclusive breastfeeding (*p* < 0.001, r = 0.13) and anger and rejection (*p* < 0.001, r = 0.19). Depression predicted lack of affection (*p* < 0.001, r = 0.25) and anger and rejection (*p* < 0.001, r = 0.54).

## 4. Discussion

In our first analysis (Table 1), the MIBS total scores were significantly higher in the mixed and formula-feeding groups than in the exclusive group. However, in our path analysis (Figure 1), which considered confounding and bidirectional relationships among factors, we did not find significant associations in either direction (breastfeeding methods to maternal–infant bonding, and vice versa). The confounding factor of parity may have caused this discrepancy in our results between comparison of groups and path analysis. In other words, the exclusive group contained more multiparous women who were likely to have lower MIBS scores. A previous study also reported that the formula milk-based group (*n* = 278) had significantly higher scores on the bonding questionnaire than did the breastfeeding group (*n* = 127) at five months postpartum (*p* = 0.04) [6]. Confounding factors such as parity may have affected their results. Regarding the relation of maternal–infant bonding with breastfeeding methods, another previous study reported that good mother–infant bonding predicted a longer duration of exclusive breastfeeding at six months [4]. As we can reasonably expect that maternal–fetal bonding changes over the course of several months, we might not find significant effects of maternal–infant bonding on breastfeeding methods at only one month postpartum. Further studies should be conducted to clarify how the bidirectional relationships between breastfeeding and maternal–infant bonding change over a few months or years after childbirth.

In this study, depression did not have significant effects on breastfeeding methods, and breastfeeding methods did not have significant effects on depression in the early postpartum period. Significant bidirectional associations (e.g., depression to early breastfeeding interruption and vice versa) were suggested by two studies with observation periods of 3 months [8] and 6 months [9] after childbirth. Although we focused on the critical period (i.e., one month after birth) when major depression and other psychiatric problems are most likely to occur [20], the main peak of prevalence rates for major postpartum depression has been reported as two months postpartum [21]. Moreover, it is inevitable that, as time passes, more women will stop exclusive breastfeeding because of depression. These results in our study therefore should be considered as a phenomenon limited to the early postpartum period.

Except for our study, only two studies have investigated the relationships between depression, maternal–infant bonding, and breastfeeding methods simultaneously. Hairston et al. collected data for the EPDS, PBQ, and breastfeeding method for postpartum mothers (*n* = 271) of infants aged 1–9 months [22]. They reported that breastfeeding was not associated with the quality of mother–infant bonding. Minamida et al. collected data (*n* = 185) for the PBQ, EPDS, and breastfeeding method for one month after childbirth [23]. They reported that breastfeeding was not associated with postpartum depression. However, they did not examine the direction of the relationship between breastfeeding and mother–infant bonding. Considering the results from Hairston et al. and our study where depression was considered as a confounding factor, breastfeeding methods may not be a central factor in mother–infant bonding.

In this study, women with higher anxiety tended to add formula-feeding to breastfeeding; this result was consistent with the findings of the Fallon et al. systematic review [10]. Regarding anxiety’s effects on breastfeeding, two studies focused on oxytocin levels and reported that women who had higher anxiety scores per the State-Trait Anxiety Inventory had lower oxytocin release during breastfeeding at eight weeks [24,25]. Accordingly, in clinics, effective childcare support and advice on childcare by nursing staff may reduce mothers’ anxiety, and increased breastfeeding continuity may result from enhanced oxytocin secretion.

Our study had several limitations that merit further discussion. First, we lacked data on the frequency and duration of breastfeeding sessions. Several notable studies have discussed the importance of frequent on-demand breastfeeding based on the infant’s feeding cues. Not on-schedule breastfeeding, but on-demand breastfeeding may be the biological norm in humans [26]. Recently, Oras et al. reported that their breastfeeding support program increased on-demand breastfeeding patterns and yielded higher self-efficacy in breastfeeding associated with exclusive breastfeeding [27]. Further studies with data on the frequency and duration of breastfeeding sessions therefore are needed to clarify relationships between breastfeeding and maternal mental health, including the relationship with bonding. Additionally, our observation period postpartum was only one month. A longer period may have produced clearer relationships among breastfeeding, maternal depression/anxiety, and maternal–fetal bonding. Furthermore, our participants were recruited from 33 obstetric institutions in Niigata Prefecture, and may not be representative of the general population of postpartum women in Japan. Finally, we could not obtain data on both endogenous oxytocin levels and synthetic oxytocin used for inducing labor and labor promotion—a topic that has received attention with regard to lactation, postpartum depression, and maternal–fetal bonding [28].

## 5. Conclusions

Our study suggests that exclusive breastfeeding is not associated with maternal-fetal bonding in the early postpartum, considering depression, anxiety, and parity. Further studies collecting data on the frequency and duration of breastfeeding sessions and employing longer observation periods are needed in order to clarify how the breastfeeding method affects maternal-fetal bonding.

## Figures and Tables

**Table 1:** Comparisons of characteristics between the exclusive breastfeeding group and the non-exclusive breastfeeding group.

	Exclusive Group	Mixed Group	Formula-Feeding Group	Statistics
Number	*n* = 1029	*n* = 945	*n* = 46	
Parity				
Primipara	*n* = 385 (41.1%) ^#^	*n* = 529 (56.5%) ^#^	*n* = 23 (2.5%)	
Multipara	*n* = 644 (59.5%) ^#^	*n* = 416 (38.4%) ^#^	*n* = 23 (2.1%)	χ^2^ = 68.5, *p* < 0.001 *
Age (year)	31.9 ± 4.76	31.2 ± 4.80	32.4 ± 4.75	F = 4.46, *p* = 0.012
HADS score (point)				
Total	10.79 ± 5.93	12.67 ± 6.68 ^a^	13.54 ± 7.56 ^b^	F = 23.7, *p* < 0.001 *
Anxiety	4.92 ± 3.60	6.08 ± 4.07 ^c^	6.57 ± 4.25 ^d^	F = 24.1, *p* < 0.001 *
Depression	5.87 ± 3.16	6.59 ± 3.44 ^e^	6.98 ± 3.83	F = 13.1, *p* < 0.001 *
MIBS score (point)				
Total	1.85 ± 2.21	2.63 ± 2.74 ^f^	2.80 ± 3.22 ^g^	F = 25.5, *p* < 0.001 *
Lack of affection	0.73 ± 1.09	1.02 ± 1.30 ^h^	1.13 ± 1.44	F = 15.4, *p* < 0.001 *
Anger and rejection	0.31 ± 0.73	0.46 ± 0.94 ^i^	0.54 ± 0.96	F = 8.59, *p* < 0.001 *

Abbreviations: HADS, Hospital Anxiety and Depression Scale; MIBS, Mother-to-Infant Bonding Scale. Age, HADS score and MIBS score are shown as the mean ± standard deviation. * The level of significance was set at *p* < 0.0063 according to the Bonferroni correction of 8 statistical tests. ^#^ The adjusted residual induced by chi-square post hoc test was more than 2.0 or less than −2.0. *p*-values by Bonferroni post hoc test after ANOVA were as follows: ^a^ *p* < 0.001, ^b^ *p* = 0.012, ^c^ *p* < 0.001, ^d^ *p* = 0.014, ^e^ *p* < 0.001, ^f^ *p* < 0.001, ^g^ *p* = 0.033, ^h^ *p* < 0.001, ^i^ *p* < 0.001 compared with the exclusive group.

## Data Availability

All relevant data are within the paper. We are not able to make the underlying data available to readers, because we do not have the permission of the ethical committees to do so.
